# Translation and initial psychometric evaluation of the Danish version of the treatment expectation questionnaire (TEX-Q)

**DOI:** 10.1186/s41687-026-01121-1

**Published:** 2026-06-20

**Authors:** Josephine Zachodnik, Louise Linding, Kasper Højgaard Thybo, Anja Geisler, Magnus Sandberg

**Affiliations:** 1 Department of Health Sciences, Lund University, Lund, Sweden; 2 Centre for Anesthesiologic Research, Department of Anesthesiology, Zealand University Hospital, Zealand Region of Denmark, Koege, Denmark; 3 Department of Clinical Medicine, University of Copenhagen, Copenhagen, Denmark; 4 Department of Digestive Diseases, Transplantation and General Surgery, Copenhagen University Hospital, Copenhagen, Denmark

**Keywords:** Treatment expectations, Questionnaire translation, Test–retest reliability, Construct validity, Spinal surgery

## Abstract

**Background:**

Patient expectations are important psychological determinants of health outcomes. The Treatment Expectation Questionnaire (TEX-Q) is a validated, multidimensional instrument for assessing treatment expectations; however, no Danish version has previously been available. This study aimed to translate and conduct an initial psychometric evaluation of a Danish version of the TEX-Q in patients undergoing spinal surgery.

**Methods:**

The TEX-Q was translated using a structured forward–backward procedure in accordance with ISPOR guidelines, including expert review by clinicians from different professional backgrounds. Consecutive adult patients scheduled for spinal surgery at a university hospital were invited to participate. The initial psychometric evaluation followed established PROM reporting standards and included assessment of feasibility, internal consistency, test–retest reliability, floor and ceiling effects, and construct validity.

**Results:**

The Danish TEX-Q demonstrated high comprehensibility and linguistic clarity, with mean item ratings ranging from 5.4 to 5.8 on a six-point Likert scale. Reliability varied across domains, with good internal consistency and test–retest reliability observed for most domains. Floor effects were negligible, while ceiling effects exceeded 15% in selected domains. Construct validity analyses showed no association between the TEX-Q total score and functional disability, while only limited domain-specific associations were observed, and a small but statistically significant positive association with quality of life.

**Conclusion:**

The Danish version of TEX-Q was successfully translated and demonstrated promising initial psychometric properties in a population of patients undergoing spinal surgery. However, variability in reliability across domains warrants cautious interpretation of domain-level results. Further studies are needed to assess structural validity and confirm the psychometric properties of the instrument in larger and more diverse clinical populations.

## Background

Patient expectations represent a key psychological determinant of health outcomes and have been identified as influential across a wide range of medical and psychological interventions [1]. As non-specific treatment factors, expectations may shape patients’ symptom appraisal, affective responses, engagement in care, and recovery trajectories [1]. In patients with musculoskeletal (muscle and skeletal) disorders and spinal problems, higher pre-treatment expectations have repeatedly been associated with improved postoperative outcomes, reduced pain intensity, and greater satisfaction with care [2–6]. These findings underscore the clinical relevance of systematically assessing expectations, particularly in surgical settings where patients’ anticipations may influence rehabilitation and long-term functional recovery.

Functional disability and quality of life are consistently related to psychological distress, with higher disability and lower quality of life being associated with increased distress. Psychological distress has previously been shown to be associated with treatment expectations, including less positive and more negative expectation profiles [7].

Existing research on patient expectations, however, is challenged by substantial heterogeneity in measurement approaches. Many studies have relied on ad hoc or locally developed questionnaires that lack theoretical grounding, standardized development procedures, and psychometric evaluation, thereby limiting interpretability and comparability across studies [8]. To address these limitations, the Treatment Expectation Questionnaire (TEX-Q) was developed as a generic, multidimensional self-report measure based on the Integrative Model of Expectations. An initial pool of 35 items was generated to capture a broad range of expectation-related constructs applicable across medical and psychological treatments. Using exploratory factor analyses and item analyses in a heterogeneous validation sample of patients undergoing different treatments (*n* = 251), the instrument was refined to a final 15-item version. This process resulted in six theoretically derived subscales reflecting key dimensions of treatment expectations: treatment benefits, positive impact, adverse events, negative impact, process, and behavioral control. The six-factor structure was subsequently supported by confirmatory factor analysis in an independent sample of patients undergoing cancer treatment (*n* = 303). Furthermore, the TEX-Q demonstrated acceptable convergent and divergent validity, as well as satisfactory two-week test–retest reliability in a subsample of psychosomatic outpatients (*n* = 28) [9]. The TEX-Q has demonstrated strong internal consistency, structural validity, and test–retest reliability. Furthermore, translation studies in English and Turkish populations have shown factor structures consistent with the original German version [7, 9].

Functional status and quality of life were selected as comparator measures, as treatment expectations are influenced by patients perceived health status, symptom burden, and overall well-being. In patients undergoing spinal surgery, higher disability and lower quality of life may shape expectations regarding treatment outcomes, making these constructs relevant for hypothesis-based construct validity assessment. However, in spine surgery, treatment expectations may not map directly into disability alone, as expectations are influenced by multiple psychological and contextual factors [10].

At present, no Danish version of the TEX-Q is available. Given that measurement properties cannot be assumed to generalize across languages or healthcare systems, rigorous translation and expert panel review are essential to ensure semantic and conceptual equivalence [11]. Moreover, psychometric evaluation is required to establish the reliability, validity, and structural integrity of the instrument within a Danish spinal surgery population. Producing a valid Danish version of the TEX-Q will facilitate high-quality Patient-Reported Outcome Measure (PROM) based research on expectations and support clinical implementation in preoperative assessment and patient-centered care.

### Aim of the study

The aim of this study is to translate and conduct an initial psychometric evaluation of a Danish version of the Treatment Expectation Questionnaire (TEX-Q) in patients undergoing spinal surgery.

## Methods

### Design

This methodological study involved the translation and initial psychometric evaluation of a Danish version of TEX-Q. The study followed a systematic process comprising forward translation, linguistic evaluation, back-translation, and assessment of test–retest reliability and construct validity.

### Translation procedure

The translation process adhered to the International Society for Pharmacoeconomics and Outcomes Research (ISPOR) guidelines for the translation and cultural adaptation of patient-reported outcome measures [12]. Following formal permission from the original author, Meike C. Shedden-Mora, three healthcare professionals (one medical doctor and two nurses) independently performed forward translations from English into Danish to ensure broad linguistic representation and clinical relevance. All forward translators were native Danish speakers with proficiency in English. These translations were synthesized into a single preliminary version through consensus discussions, with a focus on semantic clarity and conceptual equivalence. Discrepancies between translations were resolved through consensus discussions among the translators, and the final reconciled version was approved by the study investigators. Discrepant wording options were discussed during the process but were not formally logged. A native English-speaking translator, blinded to the original instrument and with no prior knowledge of the questionnaire, performed the back-translation into English. Comparison of the back-translation with the original questionnaire revealed no substantial discrepancies, supporting conceptual alignment. The original author reviewed and approved the back-translation.

Formal cognitive debriefing interviews were not conducted prior to finalization of the Danish version. However, linguistic comprehensibility and understanding were assessed in a subgroup of patients, providing patient-level feedback on item clarity and interpretation.

### Participants and setting

The present study included patients from an ongoing study on treatment expectations using the Danish TEX-Q. The parent study was approved by the Danish Data Protection Agency in the Zealand Region (REG-042-2017) and conducted in accordance with the Declaration of Helsinki [13, 14]. Patients were recruited from the Spine Center at Zealand University Hospital, Denmark, part of the Department of Orthopedic Surgery. The center provides clinical assessment and surgical treatment for a range of spinal conditions. Consecutive adult patients (≥18 years) undergoing surgery for lumbar spinal stenosis, cervical disc herniation, or other degenerative spinal disorders were included between January and December 2024. Inclusion required sufficient Danish language proficiency; no additional exclusion criteria were applied.

During the preoperative anesthetic assessment, patients received written and oral project information and provided written informed consent.

**Fig. 1:**
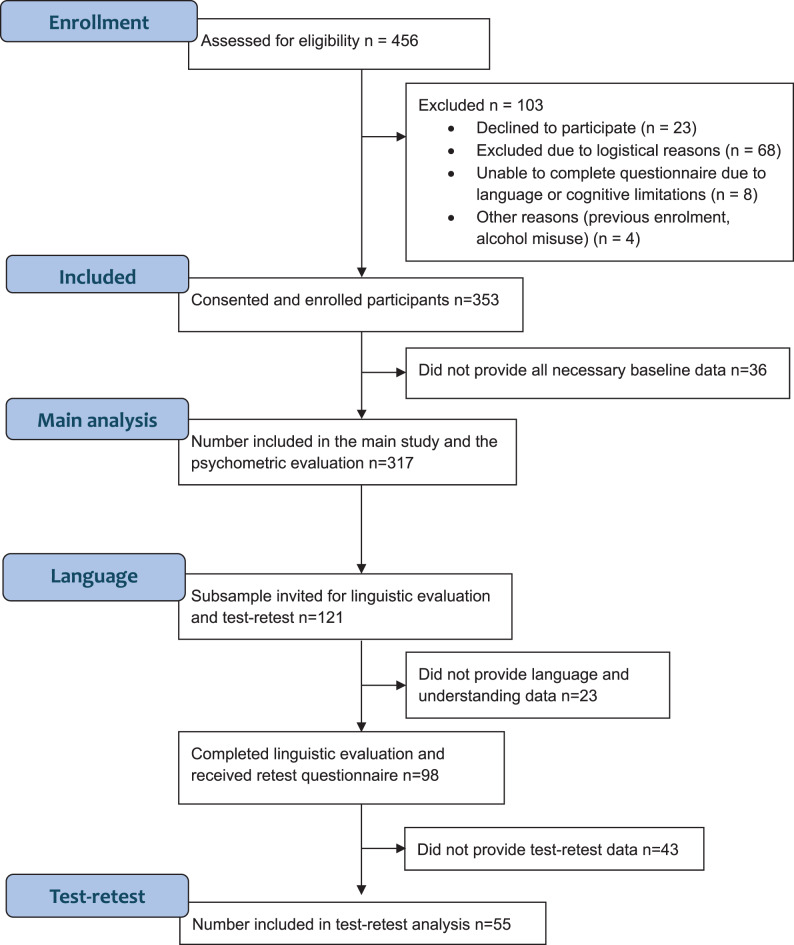
Flow chart showing the inclusion process

In the main study, a total of 353 potential participants were invited to participate. Of these, 317 were included and provided baseline data (see flowchart in Fig. 1). Those who were recruited between January and May 2024 (*n* = 121) were also invited to participate in the linguistic and the reliability parts of the study (test-retest). Of these, 98 answered the linguistic questions, and 55 completed both the initial test (baseline) and retest questionnaires. Except for the linguistic and test-rest parts, the whole sample of 317 individuals was used to investigate the psychometric properties.

### Data collection and psychometric evaluation

After providing written informed consent and a brief explanation of the study, participants completed paper-based questionnaires. Baseline characteristics included sex, age, preoperative opioid use, history of prior spinal surgery, and civil status. Test–retest reliability was evaluated in a subset of 55 patients by administering the TEX-Q twice within one week, assuming participant stability during this period, as surgery had not yet been performed. The first administration occurred during the preoperative consultation, and the retest was completed at home by the patients. To support questionnaire completion and minimize missing data, patients who completed the questionnaire at home received an SMS reminder prior to surgery to bring the completed questionnaire on the day of surgery. A one-week interval was chosen to balance the risk of recall bias against the possibility of genuine changes in treatment expectations. In this preoperative setting, baseline assessments were conducted approximately two weeks prior to surgery, and the retest was completed approximately one week before surgery, making this interval clinically relevant and ensuring that surgery had not yet been performed.

The Danish TEX-Q, administered at baseline, comprises 15 primary items and six supplementary questions across six subscales: Treatment Benefit (item 1–3), Positive Impact (item 4–6), Adverse Effects (item 7–9), Negative Impact (item 10–11), Process (item 12–13), and Behavioral Control (item 14–15) [1]. The 15 items in the TEX-Q assess treatment expectations and are rated on an 11-point numeric rating scale (0–10), with higher scores indicating stronger expectations. Items 7–11 were reverse-coded prior to analysis.

The scoring and subscale structure of the TEX-Q followed the original validation study. The total score was computed as the mean of all 15 primary items, and subscale scores were calculated as the mean of items within each subscale. Supplementary items were reported descriptively and were not included in the total score. Missing data were handled in accordance with the original TEX-Q guidelines: if more than 20% of items within a subscale were missing, the subscale score was not calculated; otherwise, the mean of available items was used [9]. For domains consisting of two items, a single missing item exceeded the predefined >20% threshold and resulted in the domain score being considered missing.

In addition, linguistic comprehension items were used to evaluate the clarity and comprehensibility of the Danish translation. These items assessed participants’ understanding of both the linguistic formulation and the intended meaning of each TEX-Q question and were rated on a six-point Likert scale ranging from 1 (“strongly disagree”) to 6 (“strongly agree”).

Participants also completed two additional validated instruments for construct validity assessment:

Oswestry Disability Index (ODI): Ten items assessing functional impairment related to lower back pain. Each item is scored on a 0–5 scale; total scores are summed and converted to a percentage (0–100), with higher scores indicating greater disability [15]. World Health Organization Quality of Life Questionnaire (WHOQOL-BREF): 26 items covering four domains: physical health, psychological health, social relationships, and environment. Items are rated on a 5-point Likert scale; domain scores are transformed to a 0–100 scale, with higher scores indicating better quality of life [16].

### Statistical analysis

Baseline characteristics were described for the total study sample and for relevant subsamples, including the test–retest group. Comparisons between groups were conducted to assess whether the test–retest subsample differed from the remaining study population.

Psychometric properties were analyzed at the item, subscale, and total score levels. Descriptive statistics were reported as means and standard deviations (SD) for continuous variables, and frequencies and percentages for categorical variables. For ordinal scale variables, such as the TEX-Q, descriptive statistics consistent with previous studies were used (i.e., means and standard deviations or medians and quartiles). Floor and ceiling effects were evaluated to assess sensitivity.

**Internal consistency,** reflecting the degree to which items within a subscale measure the same construct, was assessed using Cronbach’s alpha. Values ≥0.80 were considered good, 0.70–0.79 acceptable, and <0.70 indicative of insufficient consistency, in line with commonly used psychometric guidelines [17]. For domains consisting of two items, inter-item correlations were considered the primary measure of internal consistency, as recommended in recent validation studies of spine-related PROMs [18].

Item-level contributions to internal consistency were further examined using corrected item–total correlations (based on Pearson correlations) and Cronbach’s alpha if the item was deleted. Corrected item–total correlations ≥0.30 were considered acceptable, and substantial increases in alpha following item deletion were interpreted as indicating potential item misfit.

**Test–retest reliability**, which evaluates the stability of responses over time, was assessed at the item level using weighted kappa statistics with linear weighting, and at the subscale and total score levels using intraclass correlation coefficients (ICC) based on a two-way mixed-effects model with absolute agreement and single measurements (ICC(3,1)), in accordance with recommended guidelines [19]. Linear weighting was applied to reflect equal stepwise differences between response categories on the 11-point scale and to maintain consistency with previous TEX-Q validation studies, which treated the response scale as approximately equidistant. In addition, linear weighting provides a more conservative estimate of agreement compared with quadratic weighting. Kappa values were interpreted according to standard benchmarks [20], and ICC values ≥0.70 were considered indicative of strong reliability [21]. The sample size (*n* = 55) was deemed adequate according to established guidelines for test-retest analysis [22].

**Construct validity**, reflecting the degree to which the instrument measures the intended construct, was assessed through hypothesis testing by examining correlations between the TEX-Q total score and domain-level scores, and theoretically related outcomes (functional status (ODI) and quality of life (WHOQOL-BREF)). As no gold standard measure of treatment expectations exists, construct validity was evaluated using predefined, theoretically derived hypotheses in accordance with COSMIN recommendations. Pearson´s correlation coefficient (r) was used to assess correlations. As a sensitivity analysis, Spearman’s correlation coefficients were also calculated to account for the ordinal nature and potential non-normal distribution of the data.

It was hypothesized that higher treatment expectations would be associated with better functional status, reflected by lower ODI scores and higher WHOQOL-BREF scores. Correlation coefficients of 0.10–0.29 were considered small, 0.30–0.49 moderate, and ≥0.50 strong.

All analyses were conducted using IBM SPSS Statistics version 29.0.

The reporting followed the guidelines from COSMIN for studies on measurement properties of patient-reported outcome measures [23].

## Results

### Translation process

**Fig. 2:**
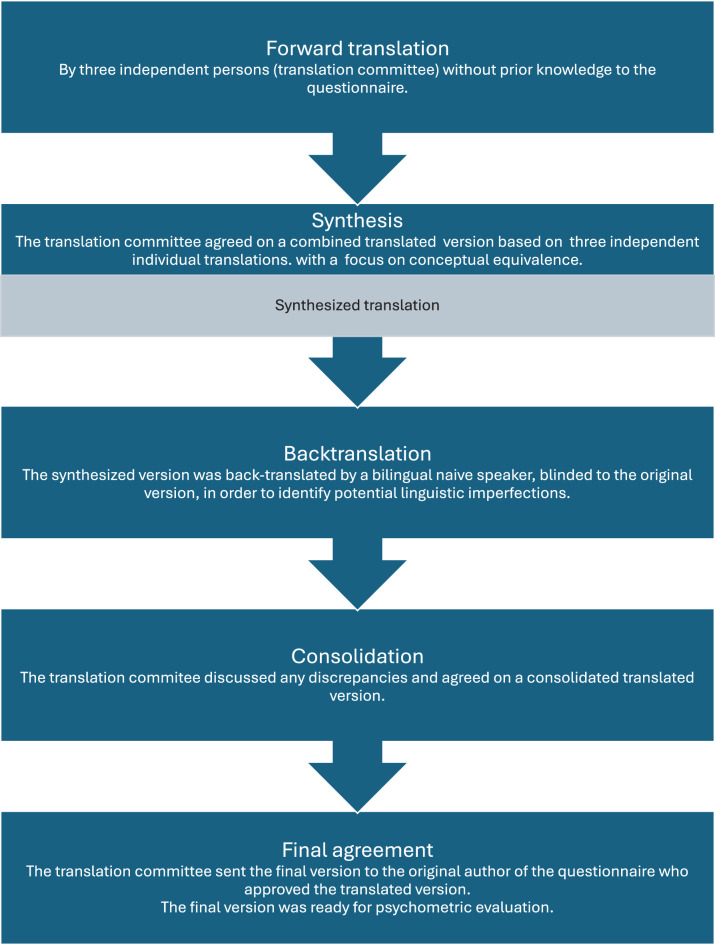
Flow chart showing the translation process (step 1–5)

The Danish version of the TEX-Q was developed using a structured forward–backward translation procedure in accordance with ISPOR guidelines (Fig. 2). The translation process was overall unproblematic, and most items achieved satisfactory semantic, conceptual, and experiential equivalence at an early stage. One item (“How much distress do you expect the treatment will cause?”) (item no. 8) required several iterations to ensure conceptual clarity and consistency with the original version. The expert panel confirmed the adequacy of the final Danish version.

### Sample characteristics and data completeness

Detailed patient characteristics of participants who completed TEX-Q at baseline (*n* = 317) are presented in Table 1. Of these, a subsample of 121 patients recruited during a specific time period was invited to participate in the linguistic evaluation and test–retest analysis. Item-level analyses of comprehensibility and language understanding were conducted in a subsample of 98 patients. For test–retest reliability analyses, 55 patients completed the questionnaire at two time points (Fig. 1). The amount of missing data was generally low across domains, ranging from 4.4% to 6.9% missing per domain at baseline (Table 2).

**Table 1:** Patients characteristics

Patients characteristics	Total study sample (n = 317)	Pre-test-only sample (n = 262)	Test-retest subsample (n = 55)
**Sex, n (%)**			
Male	144 (45.4)	121 (46.2)	23 (41.8)
Female	173 (54.6)	141 (53.8)	32 (58.2)
Age, mean (SD)	66.8 (12.2)	67 (11.8)	66.2 (13.7)
**ASA, n (%)**			
1	18 (5.6)	16 (6.1)	2 (3.6)
2	181 (57.1)	151 (57.6)	30 (54.5)
3	118 (37.2)	95 (36.2)	23 (41.8)
BMI, Mean (SD)	28.5 (5.2)	28.5 (5.2)	28.9 (5.1)
**Civil status, n (%)**			
Married	204(64.4)	168(64.1)	36(65.5)
Cohabiting	23(7.3)	21(8.0)	2(3.6)
In relationship	8(2.5)	7(2.7)	1(1.8)
Not married^a^	81(25.6)	65(24.8)	16(29.1)
**Employment status, n (%)**			
Employed	52 (16.4)	43 (16.4)	9 (16.4)
Unemployed	2 (0.6)	2 (0.8)	0 (0.0)
Retired	211 (66.6)	175 (66.8)	36 (65.5)
On sick leave	48 (15.1)	38 (14.5)	10 (18.2)
**VRS, n (%)**			
No	1 (0.3)	18 (6.9)	0 (0.0)
Mild	12 (3.8)	10 (3.8)	2 (3.6)
Moderate	64 (20.2)	52 (19.8)	12 (21.8)
Severe	237 (74.8)	197 (75.2)	40 (72.7)
Preoperative opioid use, n (%)	112 (35.3)	89 (28.1)	23 (41.8)
**Pain duration, n (%)**			
<6 months	21 (6.6)	17 (6.5)	4 (7.3)
6–12 months	88 (27.8)	74 (28.2)	14 (25.5)
1–5 years	105 (33.1)	84 (32.1)	21 (38.2)
6–10 years	35 (11.0)	32 (12.2)	3 (5.5)
>10 years	64 (20.2)	52 (19.8)	12 (21.8)
Previous spinal surgery, n (%)	124 (39.1)	107 (40.8)	17 (30.9)
WHOQOL-BREF, mean (SD)	78.9 (13.5)	83.3 (14.4)	79.6 (15.2)
ODI, mean (SD)	21.2 (8.0)	21.2 (8.1)	21.0 (7.4)

Data are presented as mean (SD) or n (%). The number of missing observations ranged from 1 to 4

Abbreviations: ASA, American Society of Anesthesiologists physical status classification; BMI, body mass index; VRS, verbal rating scale; SD, standard deviation, ODI: Oswestry Disability Index; WHOQOL-BREF: World Health Organization Quality of Life – BREF, ^a^Not married includes single, divorced, separated, or widowed

**Table 2:** Psychometric properties of total score and sub-domain scores of items in TEX-Q

	Mean (SD) (n = 317)	Missing (%) (n = 317)	Floor and ceiling effects (%) (n = 317)	Cronbach’s alpha (n = 317)	ICC (95% CI) (n = 55)
Total TEX-Q	8.8 (1.3)^a^	14 (4.4)	0.3/0.3	0.772	0.666 (0.480 to 0,795) p < 0.001
Domain 1 Treatment benefit	8.0 (1.5)	14 (4.4)	0.3/11.6	0.925	0.772 (0.635 to 0.861) p < 0.001
Domain 2 Positive impact	8.1 (1.5)	14 (4.4)	0.3/14.9	0.922	0.835 (0.732 to 0.900) p < 0.001
Domain 3 Adverse events	6.4 (2.0)	22 (6.9)	1.0/4.7	0.738	0.623 (0.423 to 0.765) p < 0.001
Domain 4 Negative impact	7.4 (2.5)	18 (5.7)	0.3/23.1	0.584	0.172 (−0.091 to 0.415) p = 0.101
Domain 5 Process	7.3 (2.0)	20 (6.3)	0.3/12.1	0.410	0.043 (−0.213 to 0.298) p = 0.373
Domain 6 Behavioural control	8.0 (2.1)	18 (5.7)	1.0/27.4	0.803	0.544 (0.339 to 0.714) p < 0.001

Data are presented as mean (SD) or %. Abbreviations: n, number; SD, standard deviation; ICC, intraclass correlation coefficient; CI, confidence interval; TEX-Q, Treatment Expectation Questionnaire. ^a^Mean of all items after inverting items 7–11

Participants included in the early subsample (January–May 2024) were comparable to those recruited later in the year with respect to baseline characteristics, and the test–retest subsample likewise did not differ meaningfully from the full study population.

### Linguistic clarity and comprehensibility of items

Linguistic clarity and comprehensibility were assessed in a subsample of the study population during the translation and cultural adaptation process. The level of Understanding of the questionnaire language was rated very high across all items, with mean scores ranging from 5.4 to 5.8 (Table 3). Patients reported high comprehensibility of the questionnaire items, with mean scores ranging from 4.9 to 5.8 on a six-point Likert scale; the lowest score was item no. 8 (Table 3).

**Table 3:** Psychometric properties of items in TEX-Q (*n* = 317 if not else stated)

	**Under-standing**^**A**^ (n = 98)	**Linguistic clarity**^**A**^ (n = 98)	**Mean (SD)**^**B**^	Missing (%)	Floor and ceiling effects (%)	Corrected item-total correlation	α if item deleted	Weighted Kappa (95% CI) (n = 55)
**Domain 1 – Treatment benefit**								
Item 1: *How much relief in your symptoms do you expect from the treatment?*	5.8 (0.6)	5.8 (0.6)	7.85 (1.7)	4.4	0.3/15.2	0.619	0.745	0.463 (0.29 to 0.63)
Item 2: *How much improvement do you expect in your ability to do your daily activities (eg, occupation, household, social life)?*	5.7 (0.9)	5.8 (0.7)	8.16 (1.6)	4.7	0.3/24.5	0.649	0.743	0.442 (0.28 to 0.61)
Item 3: *How much do you expect your health will improve as a result of the treatment?*	5.8 (0.7)	5.8 (0.7)	7.95 (1.6)	4.1	0.3/16.8	0.608	0.746	0.426 (0.26 to 0.59)
**Domain 2 – Positive impact**								
Item 4: *How much improvement do you expect in your ability to do your daily activities (e.g., occupation, household, social life)?*	5.7 (0.7)	5.7 (0.7)	7.94 (1.7)	4.1	0.7/20.4	0.593	0.745	0.400 (0.24 to 0.56)
Item 5: *How much do you expect the treatment will improve your quality of life?*	5.8 (0.8)	5.7 (0.7)	8.30 (1.6)	4.4	0.3/24.1	0.592	0.747	0.512 (0.36 to 0.68)
Item 6: *How much improvement do you expect in your ability to fulfil your day-to-day responsibilities (e.g., at home, at work, in the family)?*	5.7 (0.8)	5.7 (0.8)	8.08 (1.6)	4.1	0.7/21.1	0.645	0.743	0.334 (0.16 to 0.51)
**Domain 3 – Adverse events**								
Item 7: *To what extent do you expect risks from the treatment?*	5.6 (1.0)	5.7 (0.7)	6.22 (2.9)	5.0	2.7/12.3	0.352	0.763	0.195 (0.05 to 0.34)
Item 8: *How much distress do you expect the treatment will cause?*	4.9 (1.6)	5.4 (1.3)	5.83 (2.5)	6.6	0.2/8.4	0.354	0.762	0.146 (0.02 to 0.27)
Item 9: *To what extent do you expect side effects or other unwanted effects from the treatment?*	5.6 (0.9)	5.7 (0.8)	7.17 (2.3)	5.4	0.7/17.7	0.461	0.752	0.206 (0.06 to 0.35)
**Domain 4 – Negative impact**								
Item 10: *How much do you expect the treatment will reduce your quality of life?*	5.5 (1.1)	5.7 (0.9)	7.15 (3.3)	5.7	4.3/35.1	0.142	0.793	0.177 (0.03 to 0.32)
Item 11: *How much do you expect the treatment will limit your day-to-day responsibilities (e.g., at home, at work, in the family)?*	5.3 (1.3)	5.7 (0.8)	7.88 (2.5)	5.7	0.3/28.1	0.403	0.757	0.222 (0.08 to 0.37)
**Domain 5 – Process**								
Item 12: *To what extent do you expect the treatment procedure or process to be straight-forward?*	5.4 (1.1)	5.6 (1.0)	6.08 (3.0)	5.7	3.0/13.7	0.169	0.785	0.047 (−0.05 to 0.15)
Item 13: *To what extent do you expect to be satisfied with the treatment procedure or process?*	5.7 (0.9)	5.7 (0.8)	8.46 (1.8)	5.4	0.3/33.0	0.430	0.756	0.289 (0.13 to 0.45)
**Domain 6 – Behavioural control**								
Item 14: *To what extent do you expect to be responsible for the success of the treatment?*	5.4 (1.1)	5.6 (1.0)	7.74 (2.4)	6.0	2.0/29.9	0.134	0.782	0.316 (0.15 to 0.48)
Item 15: *To what extent do you expect your own behaviour to influence the success of the treatment?*	5.5 (1.1)	5.6 (1.0)	8.23 (2.1)	5.0	1.0/36.5	0.221	0.772	0.369 (0.20 to 0.53)

^A^Responses were rated on a 6-point Likert scale ranging from 1 (*strongly disagree*) to 6 (*strongly agree*)

^B^Items were rated on a 0–10 numeric scale, with 0 representing the worst possible outcome and 10 the best possible outcome. Item-specific verbal anchors differed according to the construct assessed. Mean of all items after inverting items 7–11

Abbreviations: SD, standard deviation; ICC, intraclass correlation coefficient

Weighted kappa coefficients are presented with 95% confidence intervals calculated using asymptotic standard errors

### Descriptive statistics and floor and ceiling effects

Mean TEX-Q domain scores ranged from 6.4 (SD 2.0) in domain *Adverse events* to 8.1 (SD 1.5) in domain *Positive impact* (Table 2). Floor effects were negligible across all domains (≤1.0%). Ceiling effects were below the predefined threshold of 15% for domains *Treatment benefit*, *Positive impact*, *Adverse events*, and *Process*, indicating no relevant ceiling effects. Higher ceiling effects were observed in domain *Negative impact* (23.1%) and domain *Behavioral control* (27.4%) (Table 2).

### Internal consistency

Internal consistency of the Danish version of the TEX-Q was assessed using Cronbach’s alpha and inter-item correlations. Cronbach’s alpha varied across the six domains, with coefficients ranging from 0.41 to 0.93. Domains *Treatment benefit* and *Positive impact* demonstrated excellent internal consistency (α > 0.90), while domains *Adverse events* and *Behavioral control* showed acceptable to good internal consistency (α > 0.70) (Table 2). For the two-item domains *Negative impact* and *Process,* inter-item correlations were considered the primary measure of internal consistency. The inter-item correlation was 0.43 for the domain *Negative impact* (Table 2). Corresponding Cronbach’s alpha values were lower (α = 0.58 and α = 0.41), as expected for two-item domains.

### Test–retest reliability

Test–retest reliability of the Danish version of the TEX-Q was assessed using intraclass correlation coefficients (ICC) based on data from 55 patients. At the domain level, domains *Treatment benefit* and *Positive impact* demonstrated good to excellent reliability, with ICC values of 0.772 (95% CI 0.635–0.861) and 0.835 (95% CI 0.732–0.900), respectively. Domain *Adverse events* showed moderate reliability (ICC = 0.623; 95% CI 0.423–0.765), while domain *Behavioral control* demonstrated moderate reliability (ICC = 0.544; 95% CI 0.339–0.714). In contrast, the domains *Negative impact* and *Process* exhibited low ICC values of 0.172 (95% CI: −0.091–0.415) and 0.043 (95% CI: −0.213–0.298), respectively (Table 2).

At the item level, test–retest agreement was assessed using weighted kappa statistics and ranged from slight to moderate across domains. Higher agreement was observed for items in domains *Treatment benefit* and *Positive impact,* with weighted kappa values up to 0.512, whereas lower agreement was found for items in domains *Adverse events, Negative impact,* and *Process*. Chi-square tests comparing response distributions between time points indicated statistically significant differences for most items; however, no consistent systematic shifts in response patterns were observed (Table 2).

### Construct validity

Construct validity was examined by assessing correlations between the TEX-Q domain and total scores, and between the TEX-Q domain and measures of functional disability (ODI) and quality of life (WHOQOL-BREF). Weak negative correlations were observed between the domains *Treatment benefit* and *Positive impact* and functional disability, indicating that more positive expectations were associated with lower disability. No significant association was observed between the TEX-Q total score and ODI.

In contrast, treatment expectations showed broader associations with quality of life. Weak to moderate positive correlations were observed for the domains *Treatment benefit*, *Positive impact*, and *Negative impact*, as well as for the TEX-Q total score, with higher expectations being associated with higher quality of life (Table 4).

**Table 4:** Construct validity of the Danish TEX-Q

	Domain 1 Treatment benefit	Domain 2 Positive impact	Domain 3 Adverse events	Domain 4 Negative impact	Domain 5 Process	Domain 6 Behavioral control	TEX-Q Total
**ODI**							
Pearson’s r	−0.192	−0.228	0.013	−0.085	0.036	0.004	−0.070
p-value	0.001	<0.001	0.824	0.148	0.534	0.949	0.226
**WHOQOL BREF**							
Pearson’s r	0.183	0.204	0.030	0.144	0.035	0.074	0.246
p-value	0.001	<0.001	0.613	0.013	0.548	0.201	<0.001

Abbreviations: ODI, Oswestry Disability Index; WHOQOL-BREF, World Health Organization Quality of Life – BREF; TEX-Q, Treatment Expectation Questionnaire

Sensitivity analyses using Spearman’s rho yielded results comparable to those obtained using Pearson correlations for associations with TEX-Q (WHOQOL: ρ = 0.200 vs. *r* = 0.246; ODI: ρ = −0.097 vs. *r* = −0.070), supporting the robustness of the findings across correlation methods.

## Discussion

The present study evaluated the Danish version of the TEX-Q regarding translation quality, reliability, and construct validity in a clinical population. The questionnaire was translated using a structured forward–backward translation procedure in accordance with ISPOR guidelines [12]. The process was largely uncomplicated, with early consensus reached for most items, indicating strong semantic and conceptual equivalence. Overall, the results demonstrate satisfactory psychometric performance of the Danish TEX-Q across most domains. Most domains demonstrated good to excellent internal consistency and test–retest reliability, with high levels of linguistic clarity and comprehensibility; however, some domains warrant further attention. Furthermore, construct validity was somewhat supported by a small observed association between treatment expectations and quality of life, whereas no meaningful association was found with functional disability. Importantly, the identified psychometric challenges were confined to specific domains rather than reflecting a general limitation of the instrument, supporting the overall usability of the Danish TEX-Q in this population.

While the Danish TEX-Q demonstrated good to excellent reliability across most domains, lower reliability estimates were observed for the domains *Negative impact* and *Process*. In contrast, both the original German and the Turkish validation studies reported acceptable to good internal consistency and test–retest reliability across all domains, including the domains *Negative impact* and *Process*, and did not identify domain-specific reliability challenges [7, 9].

Importantly, the domains *Negative impact* and *Process* comprise only two items, a scale length known to affect the stability of reliability estimates, such as Cronbach’s alpha and intraclass correlation coefficients [17]. In short scales with restricted between-subject variability, including pronounced ceiling effects, ICC estimates may become unstable and highly sensitive to small measurement errors, resulting in wide confidence intervals and reduced interpretability [17].

Important differences between study populations may further explain the observed variation in reliability estimates across validation studies. The Danish validation was conducted in a population of patients undergoing spinal surgery, with a relatively high mean age (66 years). In contrast, the Turkish validation included a younger population (mean age 43 years) consisting of patients referred for physiotherapy. Expectations related to treatment processes and anticipated negative outcomes may differ substantially between surgical and non-surgical contexts, as well as across age groups, potentially influencing both response variability and temporal stability [24, 25].

The original German validation study included heterogeneous clinical populations [9]. The main confirmation sample comprised patients undergoing treatment for various cancer diagnoses (mean age 62 years), while the test–retest reliability sample consisted of a smaller group of younger patients (mean age 41 years) receiving psychosomatic outpatient treatment [9]. In contrast, the present Danish validation was conducted in a relatively homogeneous population of older patients undergoing elective spinal surgery.

Taken together, substantial differences in age, diagnosis, and treatment context across studies suggest that the reduced reliability observed in specific domains in the Danish validation, particularly the domain *Negative impact*, may reflect population- and context-specific characteristics rather than deficiencies in the instrument itself. Domains addressing negative expectations and process-related aspects may be especially sensitive to clinical context, treatment modality, and patient experience, which could contribute to reduced temporal stability in certain populations. In addition, such domains may plausibly be influenced by norms regarding the acceptability of expressing concerns or anticipated adverse effects, potentially affecting response variability even when semantic and conceptual equivalence has been achieved through a rigorous translation process.

Methodological differences between studies, including variation in sample size for test–retest analyses and differences in the setting in which the questionnaire was completed, may further contribute to variation in reliability estimates. The clinical context in which patients complete the questionnaire, such as proximity to treatment-related information and level of interaction with healthcare professionals, may influence the stability of expectations over short time intervals.

To further explore internal consistency in these domains, inter-item correlations were examined as a complementary measure. The inter-item correlation observed for the domain *Negative impact* fell within the recommended range, suggesting an adequate association between the two items despite the lower alpha value. The restricted variability in scores was also suggested by the pronounced ceiling effects observed in these domains. When a substantial proportion of respondents endorse the highest response categories, between-subjects variability decreases, which is known to negatively affect reliability coefficients such as the ICC and internal consistency estimates [17]. Ceiling effects exceeding the recommended threshold of 15% were observed in domain *Negative impact*, suggesting limited discriminatory ability and a reduced capacity to detect change over time, which represents an important measurement property in psychometric validation studies [26]. This phenomenon may be particularly relevant in the context of preoperative expectation measures, where patients often report high expectations prior to treatment [24].

These findings suggest that methodological constraints related to scale length may contribute to the observed reduction in internal consistency; however, they are unlikely to fully account for the findings in this population [17].

Notably, similar ceiling effects were also observed in the domain *Behavioral Control*; however, this domain demonstrated acceptable internal consistency and test–retest reliability despite comprising only two items. This suggests that ceiling effects and scale length alone do not fully explain the reduced reliability observed in domain *Negative impact*. Instead, the findings point towards domain-specific characteristics, potentially related to item content, as contributing factors. Specifically, the structural characteristics include a limited number of items within the domain and restricted score variability, including ceiling effects, which may reduce the stability of reliability estimates. In addition, content-related factors, such as the abstract nature of the items and their focus on negative expectations and uncertainty, may have contributed to less consistent responses in this population.

Treatment expectations showed small but statistically significant positive associations with quality of life, which were observed at both the domain and total score level, whereas no association was observed between the TEX-Q total score and functional disability, while only limited domain-specific associations were present. This pattern aligns with the conceptual framework underlying the TEX-Q, in which treatment expectations are conceptualized as a psychological construct reflecting patients’ perceptions, beliefs, and anticipated outcomes rather than their current level of physical functioning. In the Turkish validation study, divergent validity was supported by moderate negative associations between TEX-Q scores and symptoms of anxiety and depression as measured by the Hospital Anxiety and Depression Scale, indicating that higher treatment expectations were associated with lower psychological distress [9]. Differences in the pattern and strength of associations across studies do not necessarily indicate low construct validity of the TEX-Q, as previous studies have suggested that treatment expectations may be more closely related to psychological appraisal and perceived well-being than to objective measures of functional impairment. Alternatively, the absence of an association may reflect limitations in the comparator instrument or in the study of the population’s context-specific characteristics. Although the magnitude of this association was modest, small effect sizes are commonly observed in construct validity analyses involving psychological constructions, particularly in heterogeneous clinical populations. Although Pearson correlations were used as the primary analysis to facilitate comparison with previous TEX-Q validation studies, sensitivity analyses using Spearman’s rho yielded comparable results, supporting the robustness of the construct validity findings across correlation methods. Importantly, the observed pattern of associations differed across TEX-Q domains, underscoring the multidimensional nature of treatment expectations. Taken together, these findings support the construct validity of the Danish TEX-Q and suggest that the instrument measures a conceptually meaningful dimension that is related to psychological appraisal and overall well-being, but largely independent of physical function.

### Methodological strengths and limitations

The present study has several methodological strengths. The translation of the TEX-Q followed a rigorous forward–backward procedure in accordance with ISPOR guidelines, ensuring strong semantic and conceptual equivalence. The questionnaire was evaluated in a large clinical sample, allowing for robust estimation of internal consistency and descriptive psychometric properties. In addition, test–retest reliability was assessed using both domain-level (ICC) and item-level (weighted kappa) analyses, providing a comprehensive evaluation of temporal stability.

Several limitations should be considered. Test–retest reliability analyses were based on a smaller subsample, which may have contributed to wider confidence intervals and reduced precision of reliability estimates, particularly in domains with limited between-subject score variability. However, using a smaller subsample for test–retest analyses is common practice in psychometric validation studies and is generally considered sufficient for evaluating temporal stability. Furthermore, the characteristics of the subsample did not differ significantly from those in the remaining sample. Another limitation is that no anchor question was used to confirm the stability of treatment expectations between test and retest. Although the short interval and preoperative setting were chosen to minimize the likelihood of change, expectations may still have been influenced by clinical interactions, symptom fluctuations, or personal reflections during this period. Differences in administration context between baseline and retest may also have influenced responses, as baseline questionnaires were completed in a clinical setting, whereas the retest was completed at home. For expectancy-related items, responses may be influenced by the clinical environment, proximity to healthcare professionals, or completion conditions, and this should be considered when interpreting the reliability estimates, particularly for domains with lower performance.

A limitation of the present study is that formal cognitive debriefing interviews were not conducted prior to finalization. Although linguistic comprehensibility was assessed in a subgroup of patients, this approach may have limited the depth of patient-level evaluation of semantic nuances.

A key limitation of the present study is the absence of structural validity testing. No exploratory or confirmatory factor analyses were conducted, and therefore the underlying domain structure of the Danish TEX-Q remains unconfirmed. This limits conclusions regarding the multidimensional structure of the instrument and suggests that domain-level interpretations should be made with caution. Future studies should prioritize evaluation of the factor structure in larger samples to establish the structural validity of the instrument. Accordingly, domain-level interpretations should be considered provisional until the factor structure of the Danish TEX-Q has been confirmed in future studies [10, 27].

A further limitation was that the present validation was conducted in a highly specific clinical population consisting of older patients with long-standing spinal pain undergoing elective surgery. This may limit the generalizability of the findings to other patient populations, particularly younger patients or those with different diagnoses or treatment contexts. Consequently, caution is warranted when applying and interpreting domain-level scores in other populations, as domain-specific psychometric properties may be sensitive to contextual and population-related factors. Further studies in more diverse clinical populations are therefore needed to examine the robustness and generalizability of the domain structure.

Finally, construct validity was assessed using a limited set of external measures included in the overarching clinical study and therefore not selected specifically for psychometric validation. This represents a common and pragmatic approach in clinical validation studies, particularly in applied clinical research. Future research, including additional, more targeted comparator instruments, may yield a more comprehensive assessment of the construct validity of the Danish TEX-Q.

No exploratory or confirmatory factor analyses were conducted in the present study. Given that the primary aim was translation and initial psychometric evaluation, factor analyses were not prioritized. However, given the observed domain-specific findings, particularly the reduced reliability in the domain *Negative impact*, future studies may benefit from examining the factor structure of the Danish version to further support its structural validity.

Recent spine PROM adaptation studies have emphasized comprehensive cross-cultural adaptation and extended psychometric evaluation, including structural validity and responsiveness, which should be considered in future validation of the Danish TEX-Q [27, 28].

Overall, the strengths of the study support the robustness of the findings, while the identified limitations are primarily methodological or context-specific and do not undermine the overall applicability of the instrument within similar clinical contexts.

## Conclusion

The Danish version of TEX-Q was successfully translated using a rigorous forward–backward procedure in accordance with ISPOR guidelines. This study represents an initial cross-cultural adaptation demonstrating acceptable psychometric performance across several domains in a population of patients undergoing spinal surgery. However, the domains *Negative impact* and *Process* showed weaker reliability and require further refinement or confirmation in larger Danish spine cohorts. In addition, the absence of structural validity testing limits conclusions regarding the multidimensional structure of the instrument. Further research in larger and more diverse clinical populations is needed to confirm the psychometric properties, assess structural validity and responsiveness, and evaluate the predictive value of treatment expectations for clinical outcomes.

## Data Availability

The datasets generated and/or analyzed during the current study are not publicly available due to institutional and national data protection regulations.

## Abbreviations

CI: Confidence Interval
ICC: Intraclass Correlation Coefficient
ISPOR: International Society for Pharmacoeconomics and Outcomes Research
ODI: Oswestry Disability Index
PROM: Patient-Reported Outcome Measure
SD: Standard Deviation
TEX-Q: Treatment Expectation Questionnaire
WHOQOL-BREF: World Health Organization Quality of Life – BREF
